# In Situ Hydrothermal Synthesis of Ni_1−x_Mn_x_WO_4_ Nanoheterostructure for Enhanced Photodegradation of Methyl Orange

**DOI:** 10.3390/molecules28031140

**Published:** 2023-01-23

**Authors:** Imran Hasan, Mohammed Abdullah Albaeejan, Alanoud Abdullah Alshayiqi, Wedyan Saud Al-Nafaei, Fahad A. Alharthi

**Affiliations:** Department of Chemistry, College of Science, King Saud University, Riyadh 11451, Saudi Arabia

**Keywords:** nanoheterostructure, bandgap energy, photocatalysis, charge transfer, reactive oxidants, photoabsorbption

## Abstract

The monoclinic nanocrystalline Ni_1−x_Mn_x_WO_4_ heterostructure has been successfully synthesized by the hydrothermal technique for achieving better sensitive and photocatalytic performances. Different characterization techniques such as X-ray diffraction (XRD), Fourier transform infrared spectroscopy (FTIR), ultraviolet-visible (UV–Vis), and photoluminescence (PL) spectroscopy have been employed to investigate their structural, microstructural, and optical properties. Mn-ion incorporation in the NiWO_4_ lattice reduces the particle size of the sample compared with the pure undoped NiWO_4_ sample, which has been confirmed from the transmission electron microscope image. The Tauc plot of the Ni_1−x_Mn_x_WO_4_ sample exhibits a significant decrease in bandgap energy compared with the pure undoped NiWO_4_ sample due to the quantum confinement effect. Finally, the material was explored as a photocatalyst for the degradation of methyl orange (MO) dye from wastewater under visible light irradiation. Various reaction parameters such as pH, catalyst dose, reaction time, and kinetics of the photodegradation were studied using the batch method. The results showed that the Ni_1−x_Mn_x_WO_4_ is highly efficient (94.51%) compared with undoped NiWO_4_ (65.45%). The rate of photodegradation by Ni_1–x_Mn_x_WO_4_ (0.067) was found to be 1.06 times higher than the undoped NiWO_4_ (0.062).

## 1. Introduction

The advancement in industrial productions, such as paper, dyeing, and textiles, to boost up the economy has resulted in extreme use of heavy metals, azo dyes, and other organic compounds which are discarded directly into surface and ground water [1,2]. The toxic organic and inorganic compounds that have a non-biodegradable and persistent nature pose a threat to human health and other creatures [3]. These azo dyes are very harmful in their own context, even at ng/L concentrations, and may turn into more harmful products by reacting with other chemical species in the water streams or interacting with UV light [4]. Among various azo dyes, methyl orange (MO) has been categorized as an omnipresent water pollutant which can cause diarrhea, vomiting, and skin allergies; moreover, exposure to higher concentrations can cause death [5,6]. MO and its transformation products in water can reflect certain wavelengths of sunlight and thus hinder the photosynthesis reactions of aquatic flora [7]. This process leads to a decrease in the amount of dissolved oxygen which is mandatory for the sustainability of aquatic life [8]. Thus, it is necessary for researchers to develop efficient methods to remove MO from wastewater completely without producing any secondary pollutants. Various methods currently exist, such as chlorination, ozonation, adsorption, sedimentation, and coagulation [9,10,11]. However, these methods transfer MO from one medium to another medium and produce secondary pollution. So, a green procedure, i.e., photocatalytic degradation, was taken into consideration, which constitutes a more effective and energy-efficient method as compared with its counterparts in the scope of advanced oxidation processes (AOPs). The process forms highly reactive radical species generated by the irradiation of light on a catalyst surface [12]. When interacting with pollutant molecules, these highly active radicals decompose them into small non-toxic molecules or even mineralize them into CO_2_ and H_2_O [13]. However, the conversion efficiency of this method depends upon the chemical and optical behavior of the developed catalyst material.

In recent years, vast research has been carried out to explore the novel properties of the metal oxide semiconductor nanomaterials, including TiO_2_ [13], Cu_2_O [14], ZnO [15], Fe_2_O_3_ [16], etc., and mixed-metal oxide nanomaterials, such as BiFeO_3_ [17], BiVO_4_ [18], Bi_2_MoO_6_ [19], CuWO_4_ [20], etc., as a photocatalyst for the degradation of organic pollutants. Among these, nanocrystalline metal tungstates of the general formula MWO_4_ (M = Zn, Co, Ni, Mn, etc.) have attracted the attention of scientists as an efficient semiconductor photocatalyst because of their excellent optical properties and energy bandgap [21,22]. Among various metal tungstates, NiWO_4_ has been recognized as one of the important semiconductor photocatalysts, with an energy bandgap ≂3.5 eV [23]. NiWO_4_ can be easily synthesized by the hydrothermal method, decomposition approach, and electrochemical method, but the hydrothermal route has attracted more interest to synthesize a vast number of nanomaterials due to the formation of a large crystal phase [24,25]. NiWO_4_ exhibits a monoclinic wolframite-type structure with oxygen deposited around the tungsten-associating [WO_6_] type octahedron [26]. Photocatalytic degradation involves the formation of electron-hole pairs in the catalyst under radiation, which react with surrounding water and surface-adsorbed oxygen to generate ^•^OH and ^•^O_2_^−^ radicals to degrade MO [1,5]. However, the fast recombination of these electron-hole pairs hinders the photocatalytic degradation of pollutants and poses a drawback on the efficiency of the material [27]. To overcome this drawback, various steps have been applied previously in the literature, such as composite forming [28], morphological improvement [29], doping with metal elements [30], and heterojunctions [31]. Therefore, one of the effective strategies, namely heterostructure formation, was applied in this study, which creates a methodical partition of photogenerated electron-hole pairs through band alignment to improve the photocatalytic efficiency [32]. In the present study, manganese (Mn) was doped in the crystal lattice of NiWO_4_ to improve its photocatalytic activity through changes in morphology, crystal lattice, and optical properties. One of the most important aspects of doping is to substitute the metal ion of the host material through another metal ion of smaller radius, thus creating lattice defects and oxygen vacancy, which hinders the rate of electron-hole pair recombination, reduces the energy bandgap, and thus improves the photocatalytic activity of the material towards the pollutant [26,33,34]. From the literature, the ionic radii of Ni^2+^ and Mn^2+^ are 0.069 nm and 0.065 nm; this suggests that Mn^2+^ can easily substitute some of the Ni^2+^ in the NiWO_4_ lattice and thus form a Ni_1−x_Mn_x_WO_4_ nanocomposite-type heterostructure, where x is the moles of Mn added [35]. The synthesized material was explored as a photocatalyst for the degradation of MO under a visible light source. The efficiency of the synthesized material, Ni_1−x_Mn_x_WO_4_ NC, was optimized by varying different reaction parameters, such as irradiation time, pH of the reaction medium, and catalyst dose. Finally, the kinetics of degradation was outlined depending on the photocatalytic results.

## 2. Results and Discussion

### 2.1. Material Characterization

#### 2.1.1. Crystal Structure Studies

**Figure 1:**
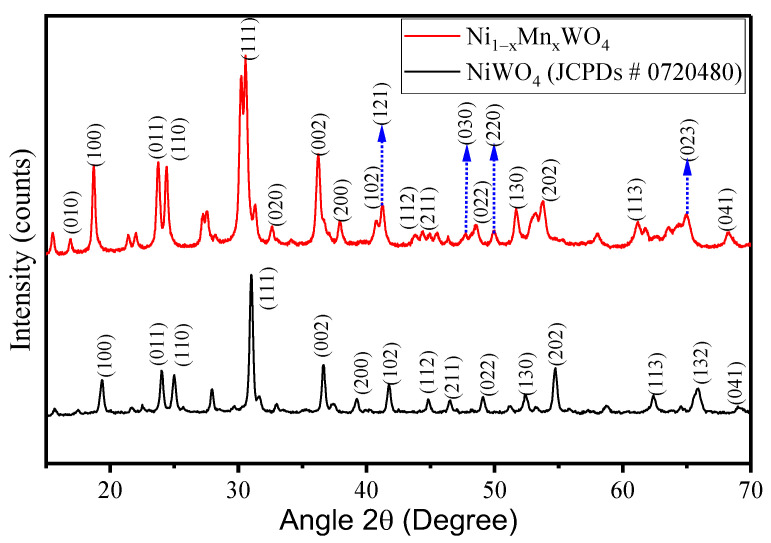
XRD spectra of NiWO_4_ (black line) and Ni_1−x_Mn_x_WO_4_ NC (red line) calcined at 600 °C.

The XRD patterns of synthesized NiWO_4_ and Ni_1−x_Mn_x_WO_4_ are given in Figure 1. The pristine NiWO_4_ exhibited the characteristic peaks at Miller indices (100), (011), (110), (111), (002), (200), (102), (112), (211), (022), (130), (202), (113), (132), and (041), which are indexed for the monoclinic wolframite structure of NiWO_4_ with standard JCPDS no. 072–0480. The XRD spectra of synthesized Ni_1−x_Mn_x_WO_4_ also exhibited maximum peaks from NiWO_4_ except for the appearance of some new Miller indices values, namely (121), (030), (220), and (023), which were found to have good agreement with the lattice structure of MnWO_4_ with standard JCPDS no. 72-0478. The XRD results suggested that Mn^2+^ has been successfully incorporated in the NiWO_4_ lattice; moreover, there is an increase in the intensity of peaks after Mn incorporation, indicating an increase in the crystallinity of material. The crystallite size was determined using the Debye–Scherrer equation given as [36]:

(1) $$D_{c}=\frac{K\lambda }{\beta \times \mathrm{Cos}\theta }$$

where λ is the wavelength of X-ray source, K is a shape factor and is usually ~0.9, θ is the corresponding angle, and β is the breadth of the observed diffraction line at its half intensity maximum. The results obtained using Equation (1) disclosed that the particle size of NiWO_4_ was 32 ± 1.05 nm, while for Ni_1−x_Mn_x_WO_4_, it was 26 ± 0.65 nm. The outcomes indicated a contraction in the crystallite size in NiWO_4_ upon Mn^2+^ incorporation, suggesting an improvement in the optical activity of the material as well.

#### 2.1.2. Functional Group Studies

**Figure 2:**
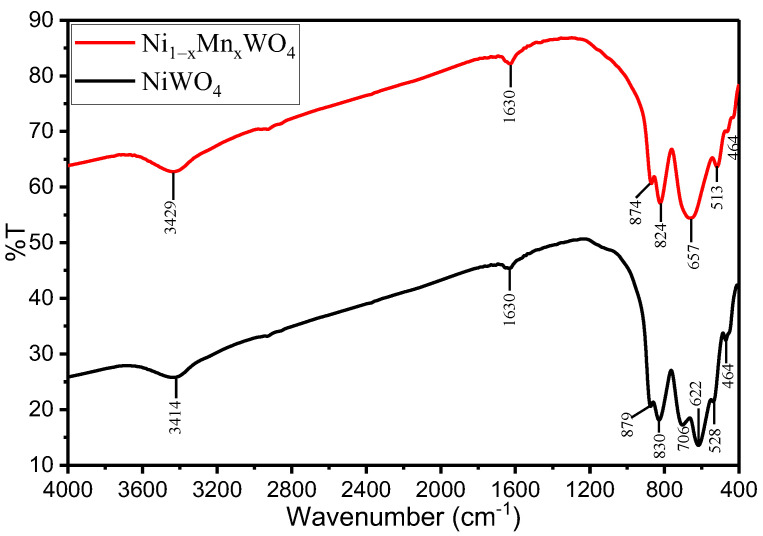
FTIR spectra of NiWO_4_ (black line) and Ni_1−x_Mn_x_WO_4_ NC (red line) calcined at 600 °C.

The functional group study of the materials was done using FTIR spectroscopy and the results are given in Figure 2. From the FTIR spectra, NiWO_4_ shows characteristic bands at 3414, 1630 cm^−1^ belonging to stretching and bending vibration bands of –OH groups (surface adsorbed water), 879 and 830 cm^−1^ antisymmetric stretching vibration bands of W–O bonds, 706, 622 cm^−1^ stretching and bending vibrations of W–O bonds in [WO_6_] octahedron, and 528, 434 cm^−1^ stretching vibrations of Ni–O bonds from the NiO_6_ octahedron [23,27]. The FTIR spectra of the as-synthesized Ni_1−x_Mn_x_WO_4_ NC exhibited the same bands with shifted values, which is due to the orbital hybridization and change of chemical environment of NiWO_4_ by Mn. The band at 706 cm^–1^ vanished completely, suggesting the acquirement of the portion associated with the [WO_6_] octahedron by Mn^2+^ [37].

#### 2.1.3. Optical Studies

**Figure 3:**
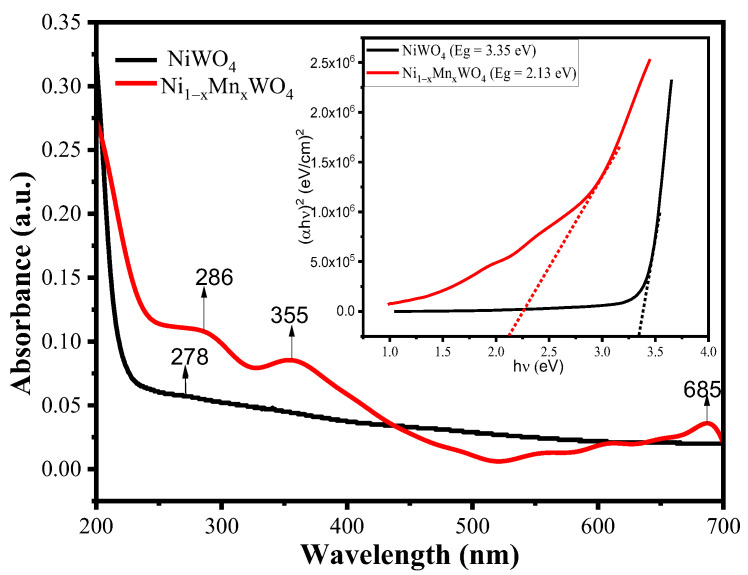
UV-Vis spectra of NiWO_4_ (black line) and Ni_1−x_Mn_x_WO_4_ NC (red line) with inset Tauc’s plot for calculating energy bandgap.

The optical properties of the synthesized material were investigated by observing the UV-Vis spectra given in Figure 3. The UV-Vis spectra of NiWO_4_ exhibited one peak of very low intensity at 278 nm, suggesting that the material is only UV-light active and the corresponding transition is due to a charge transfer process between Ni^2+^ (d–d) and octahedron [WO_6_] clusters [38]. The UV spectra of Ni_1−x_Mn_x_WO_4_ NC exhibited three peaks at 286 nm, 355 nm, and 685 nm, suggesting the UV and visible light activity of the synthesized material. So, the incorporation of Mn in the NiWO_4_ lattice enlarged its light absorbing capacity; moreover, the peaks that appeared are due to Ni^2+^ (d–d) to octahedron [WO_6_] clusters CT and secondly Ni^2+^ (d–d) to octahedron [WO_6_] clusters to Mn^2+^ CT, which increased its absorption intensity to visible light from the UV region [39]. The energy bandgap (E_g_) value of the synthesized material can be calculated using Tauc’s equation given as [40]:

(2) $$\alpha h\upsilon ={B(h\upsilon -E_{g})}^{n}$$

where B is a constant (dependent on the nature of the material), α is the absorption coefficient of the material (calculated from the absorption spectra), ν is the frequency of the radiation, h is the Plank’s constant, and E_g_ is the energy of the band gap in eV. The value of n (coefficient of transition) decides the type of energy bandgap, i.e., n = 2 value corresponds to allowed indirect energy bandgap, while n = 1/2 for allowed direct energy bandgap. Using Equation (2), the value of energy band gap (E_g_) was calculated as 3.35 eV for NiWO_4_ and 2.13 eV for Ni_1−x_Mn_x_WO_4_ NC. The contraction in Eg value upon incorporation of Mn in the NiWO_4_ lattice resulted in improved optical and photocatalytic activities.

#### 2.1.4. SEM-EDX-Mapping Analysis

**Figure 4:**
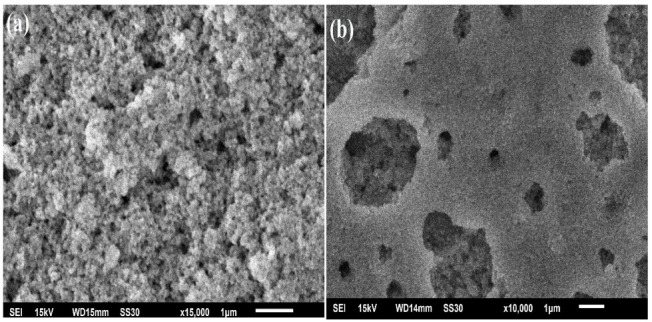

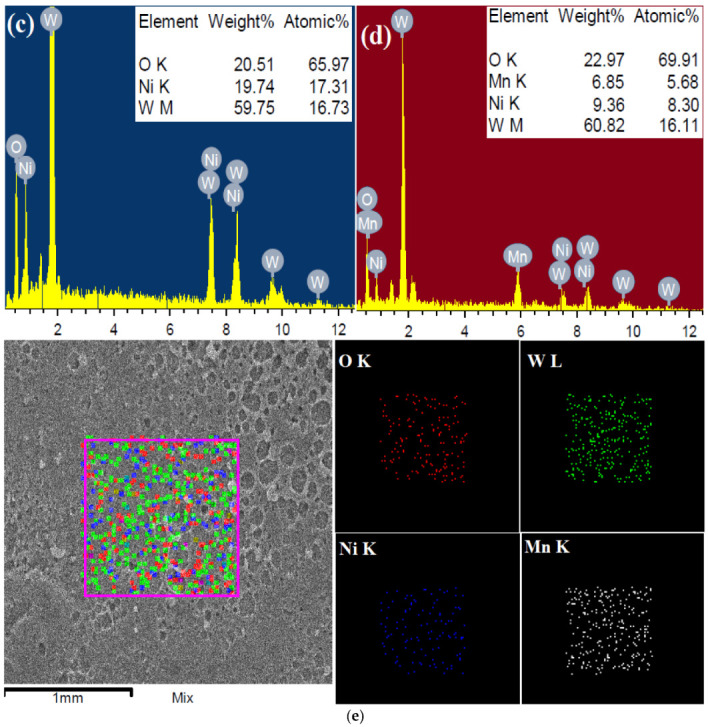
SEM-EDX image of (**a**,**c**) NiWO_4_ (**b**,**d**) Ni_1–x_Mn_x_WO_4_ NC. (**e**) Selected area mapping of elements composing the synthesized nanocomposite.

The surface morphology, elemental composition, and their distribution in the crystal lattice were assessed by SEM-EDX analysis. Figure 4a represents the SEM image of pristine NiWO_4_, which shows an aggregation of tiny interconnected particles, whereas the SEM image in Figure 4b represents a porous morphology with interconnected tiny particles. The EDX analysis given in Figure 4c,d confirms the composition of the synthesized material by weight % as O K (20.51), Ni K (19.74), and W M (59.75) for NiWO_4_, and O K (22.97), Ni K (9.36), Mn K (6.85), and W M (60.82) for Ni_1−x_Mn_x_WO_4_ NC. The EDX results suggested a ratio of 1.4:1 of Ni:Mn in the synthesized material. The elemental mapping given in Figure 4e taken from the selected marked area of Ni_1–x_Mn_x_WO_4_ NC shows a uniform distribution of O, Ni, Mn, and W in the material.

**Figure 5:**
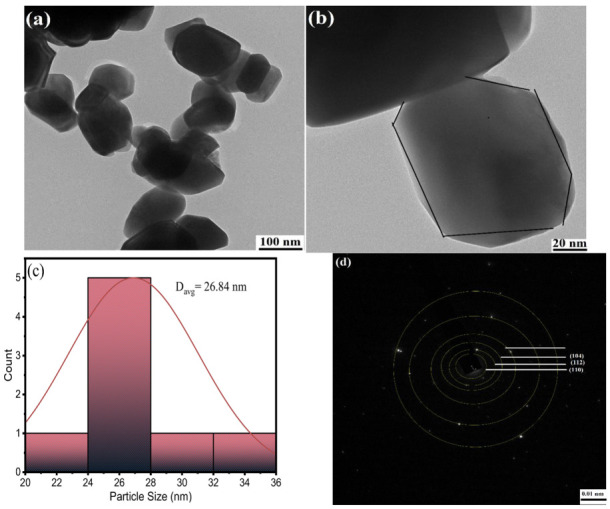
(**a**,**b**) TEM images of Ni_1−x_Mn_x_WO_4_ NC at 100 and 20 nm magnification range, (**c**) Gaussian average particle size distribution, and (**d**) SAED pattern showing the Debye–Scherer rings.

For further insight into the morphology, including crystallite shape and size, TEM-SAED analysis was used. Figure 5a,b represents the TEM image of Ni_1−x_Mn_x_WO_4_ NC, which represents the monodispersed hexagonal crystals with mitigated morphology. The Gaussian particle size distribution given in Figure 5c revealed an average crystallite size of 26.84 nm, which is found to be in good agreement with the crystallite size calculated using the Debye–Scherer equation (26 ± 0.65 nm). Figure 5d consists of the selected area diffraction pattern (SAED) of Ni_1−x_Mn_x_WO_4_ NC, with marked yellow rings belonging to the Miller indices (110), (112), and (104) indexed in the XRD pattern of Ni_1−x_Mn_x_WO_4_ NC.

### 2.2. Photocatalytic Applications

#### 2.2.1. Effect of pH

**Figure 6:**
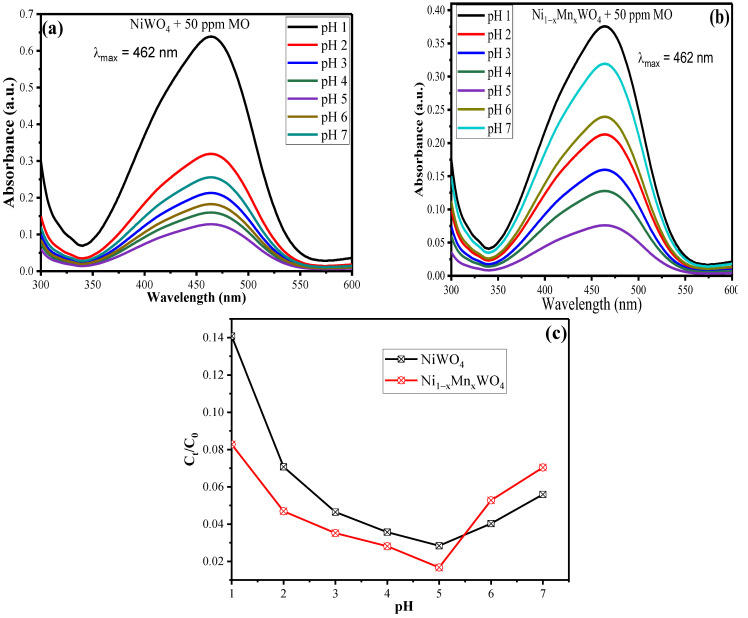
UV–Vis spectra of (**a**) NiWO_4_ (**b**) Ni_1−x_Mn_x_WO_4_ NC and (**c**) C_t_/C_0_ graph vs. pH depicting rate of MO (50 ppm) degradation for 70 min of visible light irradiation.

The pH of the reaction medium plays a vital role in controlling the rate of photodegradation of the organic pollutant by varying the charge density on the surface of the catalyst. Photocatalytic experiments were conducted by taking 20 mL of 50 ppm MO dye with 10 mg of the catalyst with varying pH values from 1–7 in cylindrical vessels inside the photocatalytic chamber associated with a tungsten lamp (150 mWcm^−2^) as the visible light source. The results obtained after completion of irradiation time are given in Figure 6a–c in which it was observed that with an increase in pH value from 1–5, the rate of degradation increases, achieving an efficiency of 97.16% for NiWO_4_ and 98.32% for Ni_1−x_Mn_x_WO_4_ NC; however, further increases in the pH value results in a decrease in the rate of degradation. This trend can be explained based on point of zero charge (pH_pzc_) value, which is found to be 5.8 for NiWO_4_ and 6.2 for Ni_1−x_Mn_x_WO_4_ NC (Figure S2). So, at pH < pH_pzc_, the surface of the catalyst is positive and is suitable to form adsorption–desorption equilibrium with a maximum number of anionic MO molecules and thus mineralize it in the presence of light utilizing photogenerated ^•^OH or ^•^O_2_^−^ radicals. However, at pH > pH_pzc_, the surface of the catalyst is negative, which will reflect the anionic MO molecules and thus a lesser number of dye molecules will be available for degradation; this is why a decrease in photocatalytic efficiency appeared at higher pH values [41]. The outcomes suggested that the Ni_1−x_Mn_x_WO_4_ NC possessed better photocatalytic efficiency as compared with pristine NiWO_4_. Thus, Mn decoration in the NiWO_4_ lattice, leading to the formation of the heterostructure, resulted in an enhancement of photocatalytic efficiency towards MO degradation.

#### 2.2.2. Effect of Catalyst Dose

**Figure 7:**
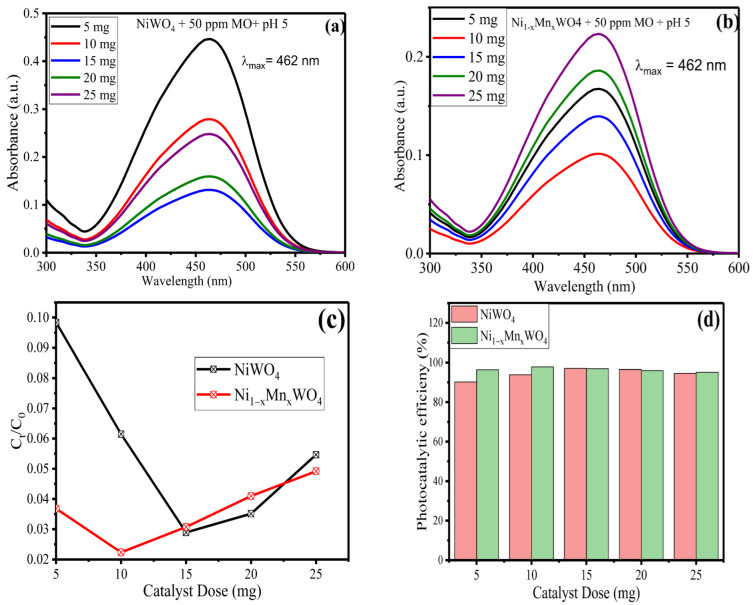
UV–Vis spectra of (**a**) NiWO_4_ (**b**) Ni_1−x_Mn_x_WO_4_ NC and (**c**) C_t_/C_0_ graph vs. (**d**) pH depicting rate of MO (50 ppm) degradation for 70 min of visible light irradiation.

Experiments were conducted by taking 20 mL of 50 ppm MO with variable catalyst dose (5, 10, 15, 20, and 25 mg) for 70 min of irradiation and results obtained are given in Figure 7a–d. From Figure 7a,b, it was observed that from 5–10 mg of catalyst dose, the absorbance value decreases, indicating the increase in rate of degradation of MO; beyond 10 mg, the rate of degradation increases as supported by Figure 7c. From Figure 7d, the maximum degradation efficiency for NiWO_4_ and Ni_1−x_Mn_x_WO_4_ NC was achieved as 93.58% and 97.46% at 10 mg of catalyst dose, respectively. Initially, with low concentration of catalyst, a greater number of surface-active sites are available to accommodate the MO molecules and thus there is a high rate of photodegradation. The decrease in the rate of photodegradation beyond a certain value of catalyst dose is due to an increase in the degree of aggregation of nanoparticles, which produces turbidity in the solution. The turbidity increases the opacity of the solution and impedes the light penetration intensity towards the catalyst surface. This phenomena decrease the rate of generation of ROS (^•^OH or ^•^O_2_^−^ radicals) and thus cause a decrease in photocatalytic efficiency [42].

### 2.3. Kinetics of Photodegradation and Effect of Irradiation Time

**Figure 8:**
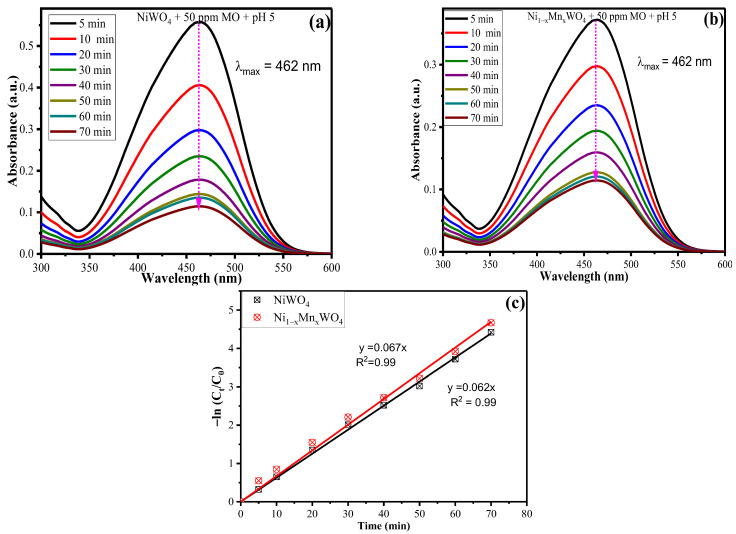
UV-Vis spectra for degradation of MO with variable irradiation time by (**a**) NiWO_4_ (**b**) Ni_1−x_Mn_x_WO_4_ NC and (**c**) linear graph of −ln (C_t_/C_0_) vs. irradiation time (t).

Figure 8a,b represents the degradation of MO in the presence of pristine NiWO_4_ and the as-synthesized Ni_1−x_Mn_x_WO_4_ NC with respect to variation in irradiation time. It can be seen from the results that with an increase in irradiation time from 5 min to 70 min, the intensity of the absorption maxima peak decreases continuously with any shift in wavelength. Thus, an increase in the rate of photodegradation of MO is observed with an increase in irradiation time until 70 min and the maximum photocatalytic efficiency was calculated as 98.79% for NiWO_4_ and 99.06% for Ni_1−x_Mn_x_WO_4_ NC, respectively. The obtained photocatalytic data was adjusted to the Langmuir–Hinshelwood (L-H) pseudo first-order kinetic model to observe the rate of degradation quantitatively. The model is mathematically given as [12,43]:

(3) $$-\ln\left(\frac{C_{t}}{C_{0}}\right)=k_{\mathrm{app}}\times t$$

where C_0_ and C_t_ are the concentration of MO at the initial state (t = 0) and specific time intervals (t) and k_app_ is the apparent rate constant which can be obtained from the slope of linear graph of -ln (Ct/C0) vs. irradiation time (t). The obtained experimental data was applied to equation (3) and the results obtained are given in Table 1 and Figure 8c. It was found that the rate of degradation of MO was higher for Ni_1−x_Mn_x_WO_4_ (0.067 min^−1^) as compared with pristine NiWO_4_ (0.062 min^−1^), which again quantitatively supports the idea of the enhancement of photocatalytic efficiency of material towards MO through Mn incorporation. The value of the correlation coefficient R^2^ = 0.99 reveals that the simulation of the L-H model to the photocatalytic data has minimum error. The half lifetime of the reaction was calculated using t_1/2_ = ln 2/k_app_, and the values obtained are 11 min for NiWO_4_ and 10.34 min for Ni_1−x_Mn_x_WO_4_ NC.

### 2.4. Detection of ROS

**Figure 9:**
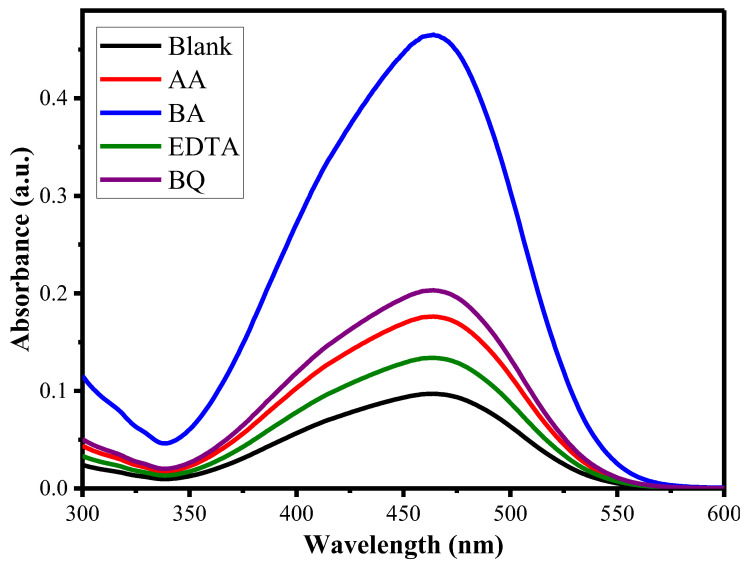
UV-Vis spectra for the scavenger test for the degradation of MO by Ni_1−x_Mn_x_WO_4_ NC.

The scavenger’s test was performed to find out the primary ROS involved in the photodegradation of MO by Ni_1−x_Mn_x_WO_4_ NC under visible light source. In the present study, some of the scavengers used include EDTA for h^+^, benzoic acid (BA for ^•^OH), acrylamide (AA for e^−^), and benzoquinone (BQ for ^•^O_2_^−^) and the results are given in Figure 9 [44]. The appearance of high absorption intensity for MO in the presence of BA suggests that the photocatalytic rate of MO is maximally inhibited by benzoic acid, which is responsible for the trapping of ^•^OH radicals. So, ^•^OH radicals are the primary ROS to degrade MO using Ni_1−x_Mn_x_WO_4_ NC.

Based on the trapping experiments, the plausible general mechanism of MO degradation is given by Equations (4–10) [1,4,8]:

(4) $${\mathrm{Ni}}_{1-x}{\mathrm{Mn}}_{x}{\mathrm{WO}}_{4}+h\nu \;\to \;{\mathrm{Ni}}_{1-x}{\mathrm{Mn}}_{x}{\mathrm{WO}}_{4}(h_{\mathrm{VB}}^{+}+e_{\mathrm{CB}}^{-})$$

(5) $${\mathrm{Ni}}_{1-x}{\mathrm{Mn}}_{x}{\mathrm{WO}}_{4}(h_{\mathrm{VB}}^{+})+{\mathrm{OH}}_{\mathrm{ad}}^{-}\;\to \;{}^{•}OH$$

(6) $${\mathrm{Ni}}_{1-x}{\mathrm{Mn}}_{x}{\mathrm{WO}}_{4}(e_{\mathrm{CB}}^{-})+O_{2}\;\to \;{}^{•}O_{2}^{-}$$

(7) $${}^{•}O_{2}^{-}+H^{+}\;\to \;{}^{•}HO_{2}$$

(8) $$2{}^{•}HO_{2}\;\to \;O_{2}+H_{2}O_{2}$$

(9) $$H_{2}O_{2}+e_{\mathrm{CB}}^{-}\;\to \;{}^{•}OH\;+{\;\mathrm{OH}}^{-}$$

(10) $${}^{•}OH\;+\;\mathrm{MO}\;(\mathrm{dye})\;\to {\;\mathrm{CO}}_{2}+H_{2}O$$

**Figure 10:**
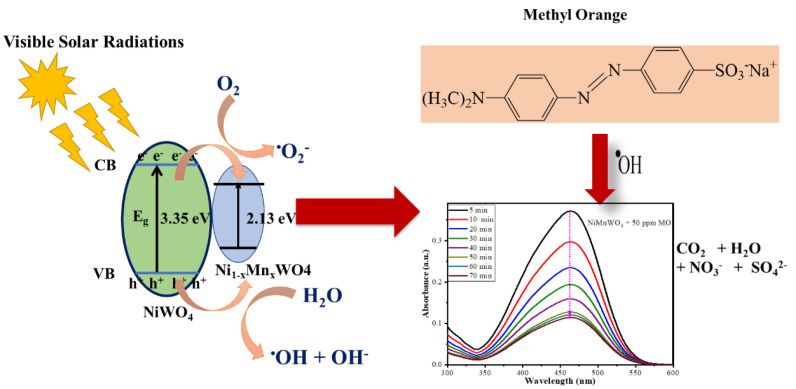
Schematic diagram showing the mechanism of photodegradation of MO by Ni_1−x_Mn_x_WO_4_ NC under visible light source.

Figure 10 represents the mechanism of photodegradation of MO by Ni_1−x_Mn_x_WO_4_ NC under visible light source. The irradiation of the photocatalyst initiates the promotion of electrons (e^−^) from the valence band (VB) to conduction band (CB) through the photoabsorption process, leaving lots of vacancies in VB, referred to as holes (h^+^), and photogenerated electrons (e^–^) in CB Equation (4). These photogenerated electrons (e^–^) in CB interact with surface-adsorbed O_2_ molecules and oxidize to superoxide radicals (^•^O_2_^−^) Equation (6). The holes interact with hydroxyl anions (OH^−^) to produce hydroxy radicals (^•^OH) Equation (5). The O_2_ molecule reacts with H^+^ ions and produces H_2_O_2_, which further reacts with e^–^ (CB) generating super reactive ^•^OH radical reaction (7–10); these are responsible for the degradation of MO under visible light source.

### 2.5. Reusability and TOC Test

**Figure 11:**
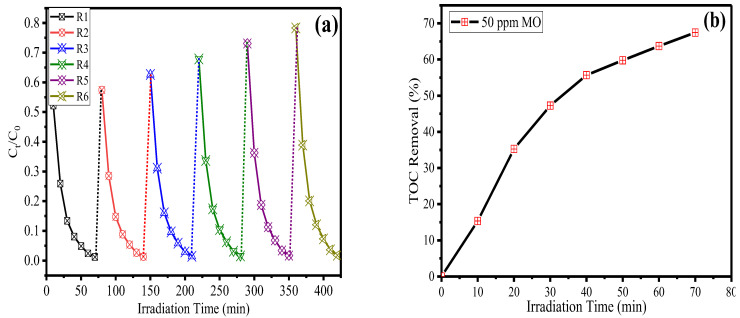
(**a**) Reusability and stability test for the synthesized Ni_1−x_Mn_x_WO_4_ NC. (**b**) TOC analysis during degradation of MO.

To check the stability of the synthesized material towards treatment of MO in wastewater, a photocatalytic test was performed in repeated mode. In cycle 1, 10 mg of the Ni_1−x_Mn_x_WO_4_ NC photocatalyst was dispersed in 20 mL of 50 ppm MO solution under optimized reaction conditions. Then, after the completion of reaction, the photocatalyst was separated out by centrifugation and the supernatant was checked using a UV-Vis spectrophotometer. The photocatalyst was washed and dried and then again dispersed in the MO solution for cycle 2. In this way, the same material underwent 6 cycles of the photocatalytic test to check its stability and reusable efficiency under the given optimized conditions towards MO. The results obtained after the photocatalytic experiment was given in Figure 11a. The outcomes suggested that the synthesized Ni_1−x_Mn_x_WO_4_ NC material is highly stable to treat MO dye as only a very small change in photocatalytic efficiency from cycle 1 (98.79%) to cycle 6 (95.23%) was observed.

Similarly, to observe the mineralization process of MO by the photocatalyst, the total organic carbon (TOC) test was taken into consideration and the results are given in Figure 11b. It was observed from the graph that as the irradiation time increases, TOC value decreases continuously, and after 70 min of irradiation, the synthesized material Ni_1−x_Mn_x_WO_4_ NC exhibits 67.45% of TOC removal, which suggests that the synthesized material is a very effective catalyst to treat MO-contaminated water under visible light source.

### 2.6. Photocurrent Measurement

**Figure 12:**
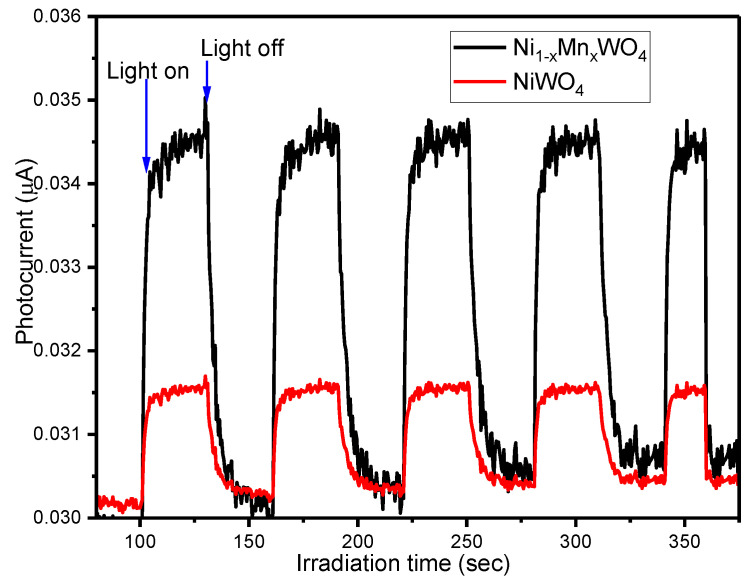
Photocurrent spectra for NiWO_4_ and Ni_1−x_Mn_x_WO_4_ NC obtained at 1.3 V.

Furthermore, to investigate the photophysical behaviors of NiWO_4_ and Ni_1−x_Mn_x_WO_4_ NC under visible light irradiation, photocurrent measurements were taken into consideration. The obtained results are given in Figure 12. It can be seen that the photocurrent response of NiWO_4_ is found to be lower than Ni_1−x_Mn_x_WO_4_ NC under visible light irradiation, which suggests that the separation efficiency of charger carriers in Ni_1−x_Mn_x_WO_4_ NC is higher than NiWO_4_. The photocatalytic experiments and optical studies are also found to be in close agreement with the photocurrent data which concludes that Ni_1−x_Mn_x_WO_4_ NC has higher photocatalytic efficiency than the NiWO_4_. The photocurrent for NiWO_4_ and Ni_1−x_Mn_x_WO_4_ NC was found to be 0.032 μA and 0.035 μA, respectively. The doping of Mn in the NiWO_4_ solid matrix leads to the creation of mobile oxygen vacancies, which prompted the separation efficiency of charge carriers; consequently, higher photocurrent was observed for Ni_1−x_Mn_x_WO_4_ NC compared with pristine NiWO_4_ [45,46].

### 2.7. Comparison with Literature

The present study was compared with the reported studies in the literature and the results are given in Table 2. It was found that the synthesized material and performed studies are purely novel as it is not reported in the literature. The material was found to be highly effective and energy efficient towards the degradation of methyl orange.

## 3. Materials and Methods

### 3.1. Chemical and Reagents

All precursor chemicals used for the synthesis, such as sodium tungstate [Na_2_WO_4_·2H_2_O] (Sigma Aldrich, St. Louis, MO, USA), nickel nitrate [Ni (NO_3_)_2_.6H_2_O] (Loba chemicals, Mumbai, India), manganese chloride [MnCl_2_.4H_2_O], and urea [CH_4_N_2_O, 99.5 %] (Merck chemicals, Rahway, NJ, USA) are of analytical grade (purity > 99%).

### 3.2. Synthesis of NiWO_4_ and Ni_1−x_Mn_x_WO_4_ Nanocomposite

The nanomaterial was synthesized by the hydrothermal process by taking the precursor mixture in a Teflon-lined autoclave at 150 °C for 24 h [51]. In a beaker, 2 moles of Ni (NO_3_)_2_.6H_2_O and 1 mole of MnCl_2_.4H_2_O were dissolved in 40 mL of deionized water and placed on a magnetic stirrer for 30 min to achieve homogeneity. After 30 min, 2 moles of Na_2_WO_4_·2H_2_O in 30 mL deionized water was added dropwise followed by the addition of 1.5 g of urea. The mixture was left on a magnetic stirrer for 3 h at room temperature (22 °C) on continuous stirring. After 3 h, the mixture was transferred to a 100 mL Teflon-lined autoclave and placed under an oven at 120 °C for 24 h to achieve complete nucleation of Mn^2+^ in the NiWO_4_ lattice. After the reaction, the mixture was taken out of the oven, cooled down and then the precipitate was collected via centrifugation (9000 rpm). The obtained material was washed with deionized water several times and ethanol (2 times) to remove unreacted entities. Finally, the material was dried in the oven at 100 °C for 4 h and then calcined at 600 °C for 3 h. Similarly, in the same way, pristine NiWO_4_ was prepared without adding MnCl_2_.4H_2_O.

### 3.3. Characterization Techniques

The synthesized NiWO_4_ and Ni_1−x_Mn_x_WO_4_ nanocomposites were characterized by various instruments to verify the synthesis of the material. The crystal structure of the material and lattice deformation upon Mn^2+^ doping was observed using powder X-ray diffraction (XRD, Bruker D8 Advance with Cu-Kα radiation, λ = 0.15418 nm, Billerica, MA, USA). The surface composition was assessed by scanning electron spectroscopy (SEM; Hitachi S-4800 Field Emission Scanning Electron Microscope, Ibaraki, Japan) and morphological information, shape and size, and their distribution in the lattice were investigated by transmission electron microscopy (TEM; JEM-2100F, Tokyo, Japan). Chemical structural information of the nanomaterial was obtained through Fourier transform infrared (FTIR; Perkin Elmer spectrum 2 ATR, Waltham, MA, USA). The optical properties of the nanoparticles were measured using ultraviolet visible spectroscopy (UV–1900 Shimadzu, Kyoto, Japan). To investigate the difference between the organic carbon (OC) and inorganic carbon (IC) during the photocatalytic reaction, a TOC analyzer (Shimadzu-00077) was used for the analysis of total organic carbon (TOC).

### 3.4. Photocatalysis Process

The photocatalytic efficiency of the as-synthesized NiWO_4_ and Ni_1−x_Mn_x_WO_4_ nanocomposites was monitored by the degradation of methyl orange (MO) under visible light irradiation using a tungsten lamp (150 mW/cm^−2^) in a photoreactor chamber. We dispersed 10 mg each of NiWO_4_ and Ni_1−x_Mn_x_WO_4_ in 10 mL of 50 ppm of MO dye solution; this was then mixed under magnetic stirring for 30 min in dark to establish the adsorption–desorption equilibrium. Finally, the mixture was taken in 20 mL cylindrical vessels (test tube, 20 mL) with a magnetic bar and irradiated in a photoreactor chamber; at a time interval, the vessels were taken out of the chamber, centrifuged to separate the catalyst from the MO solution, and then tested using a UV-Vis spectrophotometer at λmax = 492 nm to evaluate the photocatalytic efficiency as given by Equation (11):

(11) $$\mathrm{Degradation}\;\mathrm{Efficieny}\;(\%)=\left(1-\frac{C_{t}}{C_{0}}\right)\times 100$$

where C_0_ is the initial concentration of the MO solution and C_t_ is the concentration of the MO solution at specific times. A selectivity test was performed by taking 10 mg of Ni_1−x_Mn_x_WO_4_ NC with various dyes, such as bromophenol (BP), methyl orange (MO), Congo red (CR), malachite green (MG), crystal violet (CV) and methylene blue (MB). The results are given in Figure S1, which suggest that the synthesized nanocomposite material is most sensitive towards MO, showing a photocatalytic efficiency of 94.51%. Therefore, photocatalytic experiments were conducted for MO degradation by varying the reaction parameters, such as irradiation time, pH of the MO solution, catalyst dose, and concentration of the MO solution, to optimize the photocatalytic efficiency of the synthesized material. Thus, based on the outcomes of the experiments, the kinetics and mechanism of photocatalysis was predicted.

### 3.5. Reusability and TOC Test

To check the stability of the synthesized material towards the treatment of MO in wastewater, a photocatalytic test was performed in cyclic mode. In cycle 1, the photocatalyst was dispersed in the MO solution under optimized reaction conditions; after the completion of the reaction, it was separated out by centrifugation, washed and dried, and then again dispersed in the MO solution for cycle 2. In this way, the same material underwent 6 cycles of the photocatalytic test to check its stability under the given conditions towards MO. Similarly, to observe the mineralization process of MO by the photocatalyst, the total organic carbon test was taken into consideration. As the irradiation time increases, the photocatalytic efficiency also increases, suggesting an increase in the mineralization process, which will reflect in a decrease in TOC value. The TOC (%) was calculated using Equation (12):

(12) $$\mathrm{TOC}\;(\%)=\left(\frac{{\mathrm{TOC}}_{0}-{\mathrm{TOC}}_{f}}{{\mathrm{TOC}}_{0}}\right)\times 100$$

## 4. Conclusions

In the present study, we accomplished the one-pot hydrothermal synthesis of a Mn-decorated NiWO4 nanocomposite material in a Teflon-lined autoclave at 120 °C for 24 h. Various analytical and spectroscopic tests such as XRD, FTIR, UV-Vis, SEM-EDX-mapping, and TEM-SAED supported the successful incorporation of Mn in the NiWO_4_ lattice. The material Ni_1−x_Mn_x_WO_4_ showed enhanced photocatalytic degradation of MO (99.06%) as compared with pristine NiWO_4_ (93.58%) at pH 5 using 10 mg of catalyst for 50 ppm dye concentration under 70 min of visible light irradiation. The enhanced photocatalytic efficiency belongs to improved optical properties by reducing the energy bandgap (Eg) from 3.49 eV (NiWO_4_) to 3.33 eV (Ni_1−x_Mn_x_WO_4_). The SAED analysis also simulated, with XRD Miller indices data, the monoclinic wurtzite structure of the synthesized Ni_1−x_Mn_x_WO_4_ nanocomposite material. The kinetic data was best adjusted to the L-H pseudo first-order kinetic model with R^2^ = 0.99. The rate of photodegradation was found to be 1.06 times higher than the pristine nanoparticles as ^•^OH radicals play the primary reactive oxidant species. The outcomes of this study suggest that the material is highly stable and can be reusable for the mineralization of MO (67.75% TOC remove) and other organic pollutants under optimized conditions for environmental remediation without producing secondary pollution.

## Figures and Tables

**Table 1:** L-H first-order kinetic parameters for photocatalytic degradation of MO by NiWO_4_ and Ni_1−x_Mn_x_WO_4_ NC.

Catalyst	k_app_ (min^−1^)	Error	t_1/2_ (min)	R^2^
NiWO_4_	0.063	6.16 × 10^−4^	11.00	0.99
Ni_1−x_Mn_x_WO_4_ NC	0.067	1.31 × 10^−3^	10.34	0.99

**Table 2:** Comparison of the literature information with the present study.

Catalysts	Irradiation Time (min)	Light Source	Organic Pollutant	% Degradation	References
Cu-NiWO_4_	180	Visible light	Benzene	96.50	[47]
Bi-doped NiWO_4_	90	UV Irradiation	Rhodamine	86.71	[33]
rGO-NiWO_4_/Bi_2_S_3_	40	Visible light	Methyl Orange	72.00	[26]
WO_3_/NiWO_4_	80	UV Irradiation	Methylene blue	90.63	[48]
NiWO_4_-RGO	240	Visible light	o-Nitrophenol	82.00	[49]
Fe_3_O_4_/ZnO/NiWO_4_	300	Visible light	Rhodmaine B	97.90	[50]
Ni_1−x_Mn_x_WO_4_	70	Visible light	Methyl Orange	99.06%	Present study

## Data Availability

Data is contained within the article.

## Supplementary Materials

The following supporting information can be downloaded at: https://www.mdpi.com/article/10.3390/molecules28031140/s1, Figure S1. (a) UV–Vis spectra of various dyes after degradation with Ni_1−x_Mn_x_WO_4_ NC and (b) bar graph representing the % degradation values for individual organic pollutant, Figure S2. Point of zero charge for both NiWO_4_ and Ni_1−x_Mn_x_WO_4_ NC
