# Supplementary material for: In Situ Hydrothermal Synthesis of Ni1−xMnxWO4 Nanoheterostructure for Enhanced Photodegradation of Methyl Orange
Source: Molecules. 2023 Jan 23;28(3):1140. doi: 10.3390/molecules28031140 (PMC9920565; doi:10.3390/molecules28031140)
Supplement: Supplementary file 1 [file molecules-28-01140-s001.zip › molecules-2152066-supplementary.pdf]

(Supporting Information)

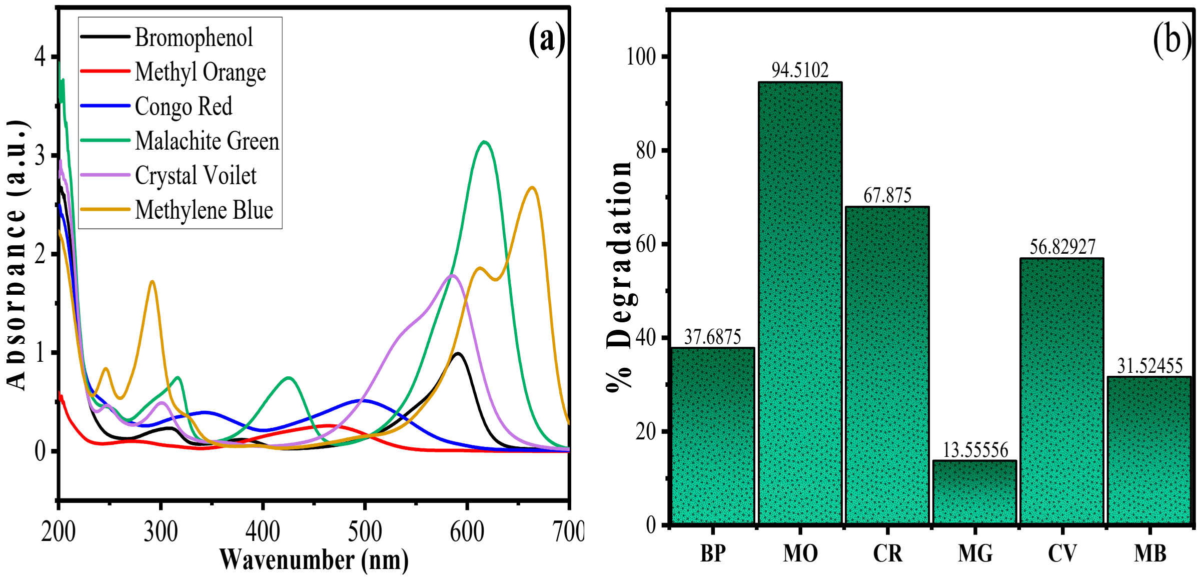

Figure S1. (a) UV–Vis spectra of various dyes after degradation with $Ni_{1-x}Mn_xWO_4$ NC and **(b)** bar graph representing the % degradation values for individual organic pollutant

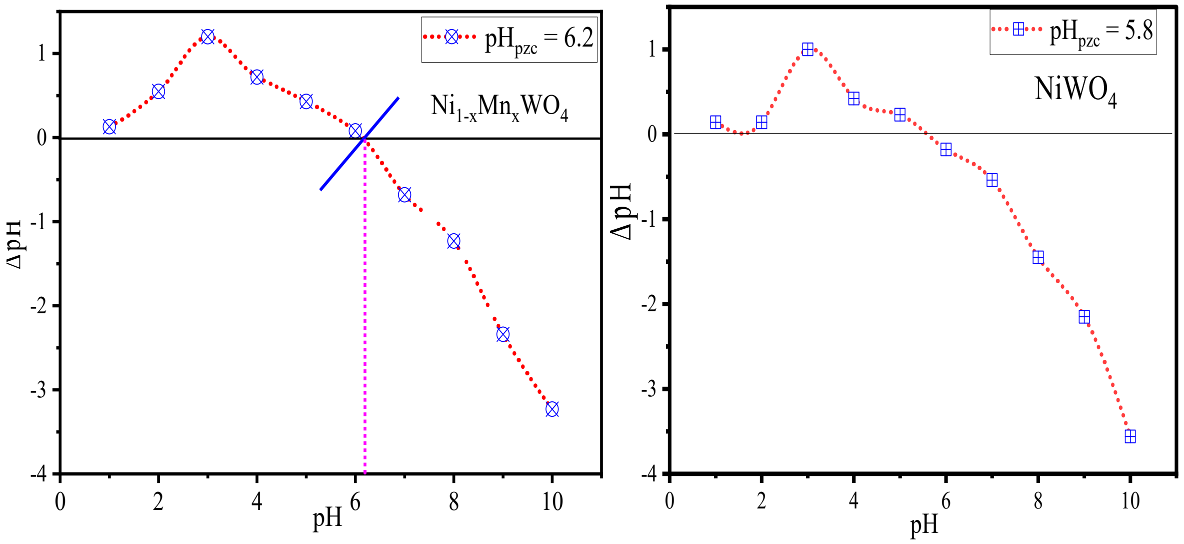

Figure S2. Point of zero charge for both NiWO4 and $Ni_{1-x}Mn_xWO_4$ NC
